# Molecular Mechanism of Blood Pressure Regulation through the Atrial Natriuretic Peptide

**DOI:** 10.3390/biology11091351

**Published:** 2022-09-14

**Authors:** Takeshi Tokudome, Kentaro Otani

**Affiliations:** 1 Department of Pathophysiology of Heart Failure and Therapeutics, National Cerebral and Cardiovascular Center Research Institute, Suita 564-8565, Japan; 2 Center for Regenerative Medicine, National Cerebral and Cardiovascular Center Research Institute, Suita 564-8565, Japan

**Keywords:** atrial natriuretic peptide, translational research, heart failure, blood pressure, molecular mechanism

## Abstract

**Simple Summary:**

Atrial natriuretic peptide (ANP) is a cardiac peptide hormone that was identified by Kangawa and Matsuo in 1984. In Japan, ANP has been used as an intravenous drug for the treatment of acute heart failure since 1995. Because ANP has a hypotensive effect, it is important to avoid excessive lowering of blood pressure when ANP is used. Recently, a compound that inhibits neutral endopeptidase, the enzyme that degrades ANP, has been developed (angiotensin receptor-neprilysin inhibitor (ARNI)). ARNI has been approved worldwide for the treatment of chronic heart failure and has been authorized in Japan as an antihypertensive drug. However, it is not understood exactly how ANP exerts its hypotensive effect. In this review, we discuss the molecular mechanism of the blood pressure-regulating effects of ANP, focusing on our recent findings.

**Abstract:**

Natriuretic peptides, including atrial natriuretic peptide (ANP), brain natriuretic peptide (BNP), and C-type natriuretic peptide (CNP), have cardioprotective effects and regulate blood pressure in mammals. ANP and BNP are hormones secreted from the heart into the bloodstream in response to increased preload and afterload. Both hormones act through natriuretic peptide receptor 1 (NPR1). In contrast, CNP acts through natriuretic peptide receptor 2 (NPR2) and was found to be produced by the vascular endothelium, chondrocytes, and cardiac fibroblasts. Based on its relatively low plasma concentration compared with ANP and BNP, CNP is thought to function as both an autocrine and a paracrine factor in the vasculature, bone, and heart. The cytoplasmic domains of both NPR1 and NPR2 display a guanylate cyclase activity that catalyzes the formation of cyclic GMP. NPR3 lacks this guanylate cyclase activity and is reportedly coupled to G_i_-dependent signaling. Recently, we reported that the continuous infusion of the peptide osteocrin, an endogenous ligand of NPR3 secreted by bone and muscle cells, lowered blood pressure in wild-type mice, suggesting that endogenous natriuretic peptides play major roles in the regulation of blood pressure. Neprilysin is a neutral endopeptidase that degrades several vasoactive peptides, including natriuretic peptides. The increased worldwide clinical use of the angiotensin receptor-neprilysin inhibitor for the treatment of chronic heart failure has brought renewed attention to the physiological effects of natriuretic peptides. In this review, we provide an overview of the discovery of ANP and its translational research. We also highlight our recent findings on the blood pressure regulatory effects of ANP, focusing on its molecular mechanisms.

## 1. Introduction

The natriuretic peptide system controls body fluid and blood pressure homeostasis via three ligands: atrial natriuretic peptide (ANP), brain natriuretic peptide (BNP), and C-type natriuretic peptide (CNP) [1,2,3,4,5,6,7,8]. ANP and BNP are cardiac hormones that induce diuresis, natriuresis, and vasodilation via the transmembrane receptor natriuretic peptide receptor 1 (NPR1) [1,2,3,4,5,6,7,8], also known as guanylyl cyclase (GC)-A. CNP is secreted from vascular endothelial cells, chondrocytes, and cardiac fibroblasts [1,2,3,5,6,7]. CNP acts via natriuretic peptide receptor 2 (NPR2) [1,2,3,5,6,7,8], also referred to as GC-B. Based on its relatively low plasma concentration compared with ANP and BNP, CNP is thought to function as both an autocrine and paracrine factor in the vasculature and the heart [9,10,11,12]. The cytoplasmic domains of both GC-A and GC-B display a guanylate cyclase activity that catalyzes the conversion of guanosine-5-triphosphate to cyclic GMP [1,2,3,5,6,8]. NPR3, which lacks the GC domain, is implicated in the metabolic clearance of natriuretic peptides and is reportedly coupled to G_i_-dependent signaling [12]. NPR3 is also known as NPR-C. Previously, Saito et al. reported that a 30 min infusion of ANP in patients with congestive heart failure reduced systemic vascular resistance, indicating that one acute effect of ANP is dilation of the resistance vessels [13]. Recently, we reported that continuous infusion of the peptide osteocrin, an endogenous ligand of NPR3, which is secreted by bone and muscle cells, lowered blood pressure in wild-type mice, suggesting that endogenous natriuretic peptides play major roles in blood pressure regulation [14]. Originally, it was thought that ANP acted via GC-A-mediated cyclic GMP production in vascular smooth muscle cells [1,15,16]; however, studies in genetically engineered mice with cell type-specific deletion of GC-A have shown that mice with a smooth-muscle-cell-specific GC-A-knockout (SMC-GC-A-KO) do not exhibit hypertension [17], whereas mice with an endothelial-cell-specific GC-A-knockout (EC-GC-A-KO) do [18]. This suggests that the endothelial ANP/BNP–GC-A system is of greater importance than its smooth muscle counterpart in long-term blood pressure regulation. However, the precise molecular mechanisms underlying endothelial-dependent blood pressure regulation and vasodilation via the ANP/BNP–GC-A system have not yet been fully elucidated.

Neprilysin is a neutral endopeptidase that degrades several vasoactive peptides, including natriuretic peptides [19]; its affinity is 3.6-fold greater for ANP than for BNP [20]. The increased worldwide clinical use of angiotensin receptor-neprilysin inhibitor (ARNI) for the treatment of heart failure has brought renewed attention to the physiological effects of ANP. Because ANP has a hypotensive effect because of its vasodilatory property, the blood pressure of patients taking ARNI should be carefully observed so as to avoid excessive hypotension. Thus, it is important from a clinical viewpoint to clarify the detailed molecular mechanisms of acute and long-term blood pressure regulation by ANP.

This review provides an overview of the discovery, translational research, and blood pressure-regulating effects of ANP, including our recent findings.

## 2. Discovery of ANP and Its Receptor

With the invention of the electron microscope, the presence of secretory granules in mammalian atrial myocytes was reported in 1964 [21]. However, the physiological significance of this finding was not highlighted until over 15 years later. In 1981, de Bold et al. reported that the intravenous administration of a rat atrial extract to another rat produced marked diuresis and hypotension [22]. Furthermore, Currie et al. reported in 1983 that gel filtration chromatography-treated samples of human, rat, and pig atrial extracts relaxed the rabbit aorta and chick rectum, and produced natriuresis in rats [23]. They also reported that boiling atrial extracts for 10 min did not abolish their aortic relaxant activity, whereas trypsin treatment did, which indicated that the intra-atrial natriuretic factor was a small-molecular weight protein [23]. During the same period, Matsuo et al. had been searching for novel, brain-derived peptide hormones. They were interested in the paper by Currie et al. and sought to identify natriuretic factors in the human atria. They successfully determined the amino acid sequence of human mature ANP (α-ANP) and confirmed that α-ANP has a potent diuretic and natriuretic activity, publishing their results in a paper titled “Purification and complete amino acid sequence of alpha-human atrial natriuretic polypeptide (α-hANP)” in 1984 [24]. Although human α-ANP is composed of 28 amino acids, they showed that there are three molecular forms of ANP, specifically α-ANP (molecular weight ~3000 Da), β-ANP (molecular weight ~5000 Da), and γ-ANP (molecular weight ~13,000 Da) [24]. Subsequent studies confirmed that β-ANP is an inverted parallel dimer of α-ANP and that γ-ANP is a precursor of α-ANP. Kangawa et al. published the amino acid sequences of both molecules in 1985 and also showed that the diuretic activities of β-ANP and γ-ANP were approximately 25% and 15%, respectively, of that of α-ANP [25]. Note that Flynn et al. discovered rat ANP (atrial natriuretic factor (ANF)) at about the same time as the discovery of human ANP by Kangawa and Matsuo et al., and reported its 28-amino-acid sequence in their paper [26]. The gene encoding ANP contains three exons, and its transcript is translated to a 151-amino-acid precursor, preproANP. The 25-amino-acid signal peptide is then removed, yielding the 126-amino-acid proANP (γ-ANP), which is the tissue form of ANP [4]. ProANP (γ-ANP) is thought to be proteolytically converted to α-ANP (the mature biologically active form of ANP) by the transmembrane enzyme corin during its secretion from the heart [27,28].

**Figure 1:**
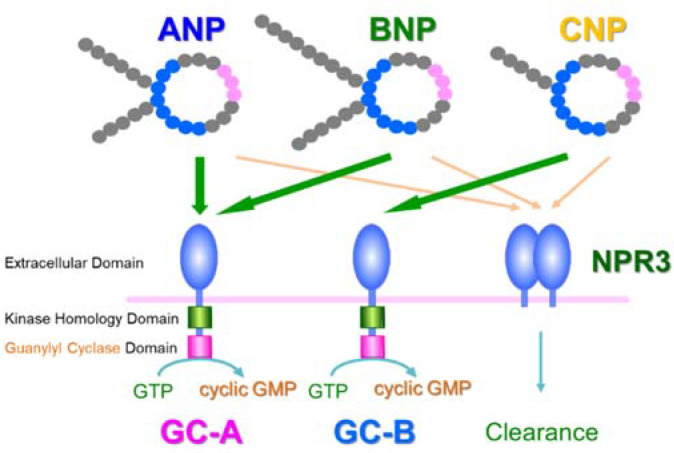
Natriuretic peptides and their receptors. ANP—atrial natriuretic peptide; BNP—brain natriuretic peptide; CNP—C-type natriuretic peptide; GC-A—guanylyl cyclase-A; GC-B—guanylyl cyclase-B; NPR3—natriuretic peptide receptor 3.

The identification of a receptor for ANP was necessary in order to fully determine its physiological significance. Hirata et al. reported in 1984 that stimulating the cultured vascular smooth muscle cells with ANP increased intracellular cyclic GMP [29]. Then, in 1989, Chinkers et al. cloned the cDNA of GC-A, a receptor for ANP and BNP [30]. They isolated partial-length GC sequences from a human cDNA library using the cDNA of sea urchin membrane-type GC as a probe [30]. This enabled them to identify the site encoding full-length GC from a rat brain cDNA library and to confirm that the gene transfer of the cDNA into COS-7 cells increased intracellular cyclic GMP upon ANP stimulation [30]. Furthermore, Schulz et al. reported in 1989 that GC-B is a membrane-bound GC that differs from GC-A [31]. Although it is now known that GC-B is the receptor for CNP, CNP was not identified until Sudoh et al. reported it in 1990 [32]. Thus, the receptor (GC-B) was found one year earlier than the ligand (CNP). Figure 1 shows the ligands and receptors of the natriuretic peptides. Natriuretic peptides are removed from the blood by binding to NPR3, a clearance receptor; because NPR3 lacks an intracellular GC domain, its binding does not increase the intracellular cyclic GMP concentration [1,5,6].

## 3. Translational Research of ANP

Saito et al. reported in 1987 that the intravenous infusion of ANP markedly improved hemodynamics in patients with heart failure [13]. In their study, the intravenous administration of ANP at 0.1 µg/kg/min for 30 min in patients with New York Heart Association III or IV heart failure decreased pulmonary artery wedge pressure by an average of 13.7 mmHg and increased the stroke volume index by an average of 7.8 mL/m^2^ [13]. ANP administration also markedly reduced systemic vascular resistance in heart failure patients [13]. Because ANP has no obvious inotropic effect, the increase in stroke volume index was presumably secondary to a decrease in systemic vascular resistance. The authors provided a very suggestive note in the discussion of their paper: “The decreased total systemic resistance observed in this study indicates that ANP dilates resistance vessels” [13]. Based on these translational studies, an α-human ANP product was approved in Japan in 1995 for the treatment of acute heart failure.

ANP administration was also found to improve the prognosis of patients with acute myocardial infarction. A multicenter, randomized, placebo-controlled, clinical trial named J-WIND enrolled patients with an initial acute myocardial infarction that occurred within 12 h of symptom onset [33,34]. The infusion of ANP for 3 days reduced the infarct size by 14.7% compared with the placebo treatment. Moreover, compared with patients who received the placebo, those treated with ANP had a higher left ventricular ejection fraction at the chronic stage after myocardial infarction. Although ANP administration did not improve the survival rates or the incidence of cardiovascular events, the incidences of cardiac death and hospital readmission due to heart failure were significantly reduced in ANP-treated patients compared with the controls [33,34].

Meanwhile, omapatrilat, which inhibits both neprilysin and the angiotensin-converting enzyme, was developed but was not clinically approved, owing to the appearance of angioedema in the clinical trial phase [35]. Bradykinin is also a substrate of neprilysin [19], and the synergistic effect of angiotensin-converting enzyme inhibition on bradykinin degradation may have caused the angioedema. Because angiotensin II is also a substrate of neprilysin [19], the clinical application of neprilysin inhibitors requires simultaneous inhibition of the renin-angiotensin system (RAS). ARNI inhibit RAS through angiotensin II receptor antagonism, which probably prevents the overproduction of bradykinin. The PARADIGM-HF study evaluating the superiority of ARNI over enalapril in heart failure patients with a reduced left ventricular ejection fraction was terminated early due to the significant prognostic value of the ARNI [36], and this resulted in its approval.

## 4. GC-A Expression in Blood Vessels

**Figure 2:**
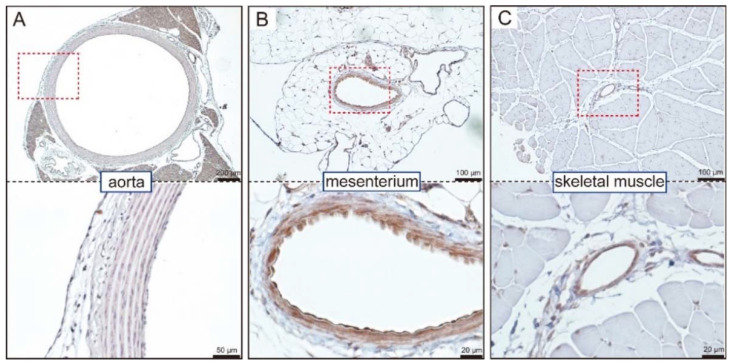
GC-A is abundantly expressed in rat small arteries and arterioles. Localization of GC-A in the aorta (**A**), mesenterium (**B**), and skeletal muscle (**C**) was examined by immunohistochemistry in 4-week-old male Sprague-Dawley rats. The region within the red dotted box in the upper part of each panel is magnified in the bottom part of each panel. This figure is a reconstruction of the figure in our original article [37].

Now that ARNI is applied clinically, elucidating the molecular mechanisms of blood pressure regulation by ANP is an important issue. However, it remains somewhat unclear how ANP exerts its acute and long-term hypotensive effects. Because few reports have examined the tissue expression of GC-A, we performed immunohistochemical staining of GC-A in rat tissues using a mouse monoclonal antibody to GC-A, paying particular attention to blood vessels (Figure 2) [37].

There was minimal expression of GC-A in the aortic endothelium, but slight positive staining was seen in aortic smooth muscle cells (Figure 2A). In contrast, the mesenteric arteries, which are representative resistance vessels, exhibited an abundant expression of GC-A in both the endothelium and smooth muscle cells (Figure 2B). No expression of GC-A was observed in the skeletal muscle cells, but there was significant expression in the interstitial vessels and capillaries (Figure 2C). These results revealed that the expression of GC-A is abundant in both the endothelium and smooth muscle cells in resistance vessels (arterioles and small arteries), but it is minimal in smooth muscle cells in conduit vessels such as the aorta. The abundant expression of GC-A in resistance vessels is consistent with a previous report [38].

## 5. Mechanism of Blood Pressure Reduction by Intravenous ANP Infusion

**Figure 3:**
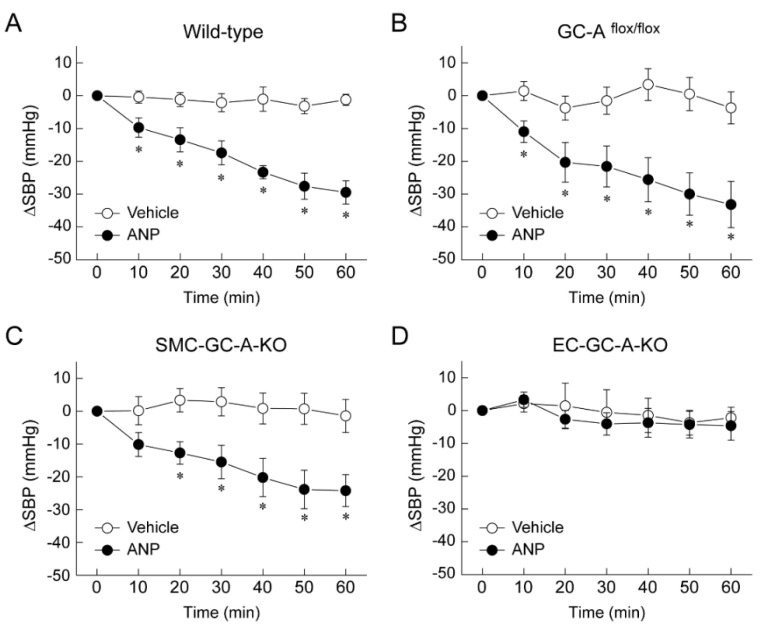
GC-A in vascular endothelial cells contributes to hypotension induced by ANP infusion. Intravenous infusion of ANP (10 μg/kg/min) significantly lowered systolic blood pressure (SBP) compared with the administration of vehicle (saline) in wild-type mice (**A**), GC-A floxed mice (GC-A^flox/flox^), (**B**), and smooth-muscle-cell-specific GC-A knockout mice (SMC-GC-A-KO), (**C**), but not in endothelial-cell-specific GC-A knockout mice (EC-GC-A-KO), (**D**). * *p* < 0.05 vs. vehicle. This figure is a reconstruction of the figure from our original article [37].

**Figure 4:**
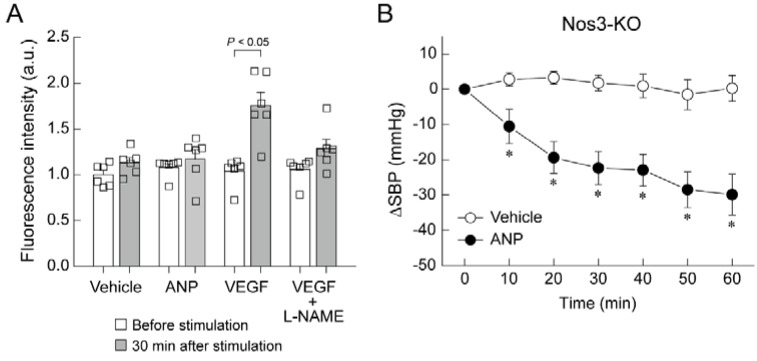
ANP-induced hypotension is independent of nitric oxide. (**A**) The measurement of nitric oxide in human umbilical vein endothelial cells (HUVECs). HUVECs were loaded with DAF-2 DA (a fluorescent nitric oxide probe) and then treated with vehicle (PBS), ANP, VEGF, or VEGF + L-NAME. Nitric oxide production was measured as cellular fluorescence (arbitrary units). Values are expressed relative to the fluorescence intensity before vehicle treatment. (**B**) Intravenous infusion of ANP (10 μg/kg/min) significantly lowered systolic blood pressure (SBP) compared with the administration of vehicle (saline) in Nos3-knockout mice (Nos3-KO). * *p* < 0.05 vs. vehicle. This figure is a reconstruction of the figure from our original article [37].

We generated our own GC-A floxed mice, then crossed them with SM22α-Cre-Tg mice to generate smooth-muscle-cell-specific GC-A-knockout mice (SMC-GC-A-KO), and with Tie2-Cre-Tg mice to generate endothelial-cell-specific GC-A-knockout mice (EC-GC-A-KO). Wild-type mice, GC-A floxed mice, SMC-GC-A-KO, and EC-GC-A-KO received ANP at 10 µg/kg/min under urethane–chloralose anesthesia with ventilator control and rectal temperature maintenance at 38 °C. Blood pressure was continuously monitored for 60 min by inserting a pressure catheter through the right carotid artery; the vehicle group received saline solution. A significant decrease in systolic blood pressure was observed in wild-type and GC-A floxed mice after ANP administration (Figure 3A,B) [37]. Unexpectedly, a similar decrease in systolic blood pressure was observed in SMC-GC-A-KO mice (Figure 3C) [37]. In contrast, EC-GC-A-KO mice exhibited no significant decrease in systolic blood pressure following ANP administration (Figure 3D) [37]. These results suggest that vascular endothelial GC-A plays an important role in the acute hypotensive effects of ANP. What is the mechanism through which the ANP-GC-A system in the vascular endothelium causes a decrease in blood pressure? We examined the association between the ANP-GC-A system and the nitric oxide system using cultured human umbilical vein endothelial cells (HUVECs) in experiments in vitro. We used DAF-2 DA fluorescent probes to detect nitric oxide. As shown in Figure 4A, stimulation of HUVECs with vascular endothelial growth factor (VEGF) resulted in a significant increase in fluorescence intensity, which was almost completely suppressed by the concurrent administration of L-NAME, a nitric oxide synthase inhibitor [37]. Meanwhile, stimulation of HUVECs with ANP (100 nM) did not alter the fluorescence intensity of DAF-2 DA at all [37], suggesting that ANP has little effect on the production of nitric oxide in cultured vascular endothelial cells. In addition, the intravenous administration of ANP to endothelial-type nitric oxide synthase (NOS3)-deficient mice resulted in a marked decrease in systolic blood pressure (Figure 4B) [37]. These results suggest that intravenously administered ANP induces endothelial GC-A-dependent hypotension by a mechanism independent of the nitric oxide system. Consistent with this, Frees et al. recently reported that ANP and BNP dilate human intrarenal arteries that have been isolated from renal cancer patients, and that the vasodilating effect of BNP is independent of nitric oxide [38].

**Figure 5:**
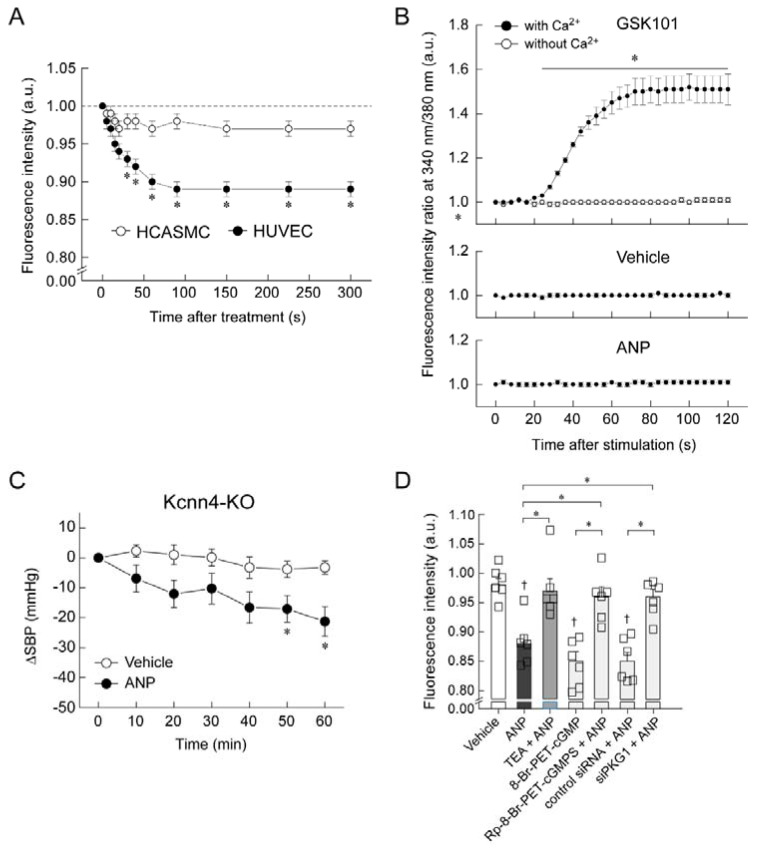
ANP causes hyperpolarization of cultured vascular endothelial cells in a K^+^ channel-dependent manner via the GC-A–PKG1 signaling pathway. (**A**) ANP significantly decreased the membrane potential of human umbilical vein endothelial cells (HUVECs) compared with that in the human coronary artery smooth muscle cells (HCASMCs). Changes in the membrane potential were evaluated by monitoring the cellular fluorescence intensity. Values are expressed relative to the intensity at time 0. * *p* < 0.05 vs. HCASMCs. (**B**) ANP had no effect on the intracellular calcium concentration of HUVECs. Fura-2-AM-loaded HUVECs were stimulated with or without ANP, and cellular fluorescence was monitored for 120 s. GSK101 (TRPV4 agonist) was used as the positive control. * *p* < 0.05 vs. without Ca^2+^. (**C**) Intravenous infusion of ANP (10 μg/kg/min) significantly lowered systolic blood pressure (SBP) compared with vehicle (saline) administration in intermediate/small-conductance K_Ca_ (Kcnn4) knockout mice. * *p* < 0.05 vs. vehicle. (**D**) ANP caused hyperpolarization of cultured HUVECs in a K^+^ channel-dependent manner via the NPR1–PKG1 signaling pathway. HUVECs were treated with or without ANP (100 nM) or 8-Br-PET-cGMP (PKG1 activator; 200 μM) in the presence or absence of a non-selective K^+^ channel blocker TEA (tetraethylammonium; 10 mM), PKG inhibitor (Rp-8-Br-PET-cGMPS; 200 μM), or PKG1 siRNA for 300 s. * *p* < 0.05. ^†^ *p* < 0.01 vs. vehicle. This figure is a reconstruction of the figure from our original article [37].

Similar to nitric oxide, endothelium-derived hyperpolarizing factor (EDHF) is an endothelium-dependent vasodilator [39,40,41]. Although nitric oxide is a major dilator in large conduit vessels in the vasculature, EDHF is the major dilator in resistance vessels, which define blood pressure and organ blood flow [39,40,41]. Because GC-A was abundantly expressed in resistance vessels in our study (Figure 2) and the endothelial ANP-GC-A system caused hypotension in a nitric-oxide-independent manner (Figure 4), we hypothesized that ANP causes hypotension by hyperpolarizing the vascular endothelium and dilating resistance vessels. We analyzed the ANP-induced change in the membrane potential of cultured vascular cells (HUVECs and human coronary artery smooth muscle cells (HCASMCs)) using DiBAC4, a membrane potential-reactive dye [37]. Three hundred seconds after stimulation with ANP, DiBAC4 fluorescence was significantly lower in HUVECs than in HCASMCs (Figure 5A), indicating greater ANP-induced membrane hyperpolarization in the former than in the latter. We then investigated the mechanism of ANP-induced hyperpolarization of the endothelial cell membranes by in vitro and in vivo studies. In general, increasing the intracellular Ca^2+^ concentration of endothelial cells and opening small- and intermediate-conductance K_Ca_ channels induced endothelial hyperpolarization [39,40,41].

We examined whether ANP affected the intracellular Ca^2+^ concentration in HUVECs by using the Ca^2+^-reactive dye Fura-2-AM. GSK101 (a transient receptor potential vanilloid 4 agonist) was used as the positive control. In GSK101-treated cells, the ratio of fluorescence intensity at 340/380 nm increased over time in the presence of Ca^2+^ (Figure 5B). However, the fluorescence intensity ratio in cells treated with vehicle or ANP remained unchanged, even in the presence of Ca^2+^ (Figure 5B) [37]. In an in vivo study that investigated whether small- and intermediate-conductance K_Ca_ channels are involved in ANP-induced hypotension, we used mice with knockout of the potassium intermediate-/small-conductance calcium-activated channel, subfamily N, member 4 (Kcnn4) [37]. The infusion of ANP into Kcnn4-KO mice significantly lowered systolic blood pressure compared with vehicle infusion (Figure 5C), indicating that small- and intermediate-conductance K_Ca_ channels are not needed for ANP to induce its hypotensive effect. To further investigate the contribution of potassium channels to ANP-induced hyperpolarization of HUVECs, we examined whether this hyperpolarization was inhibited by a potassium channel blocker (Figure 5D) [37]. Tetraethylammonium (a non-selective potassium channel blocker) completely blocked the ANP-induced decrease in DiBAC4 fluorescence in HUVECs. These results indicate that ANP-induced HUVEC hyperpolarization is likely to be mediated by potassium channels other than K_Ca_. We further examined whether the observed ANP-induced hyperpolarization of HUVECs was dependent on the cGMP–protein kinase G (PKG) pathway (Figure 5D). Moreover, 8-Br-PET-cGMP (a cGMP analogue) mimicked the ANP-induced decrease in DiBAC4 fluorescence in HUVECs. In contrast, Rp-8-Br-PET-cGMPS (a PKG inhibitor) significantly inhibited the ANP-induced decrease in DiBAC4 fluorescence. Furthermore, transfection with siRNA targeting PKG1 (siPKG1) significantly blocked the ANP-induced decrease in DiBAC4 fluorescence. Together, these findings indicate that the observed ANP-induced hyperpolarization of HUVECs was dependent on the cGMP–PKG pathway.

## 6. Regulator of G-Protein Signaling 2 (RGS2) Plays a Key Role in the Acute Hypotension Effect of ANP

**Figure 6:**
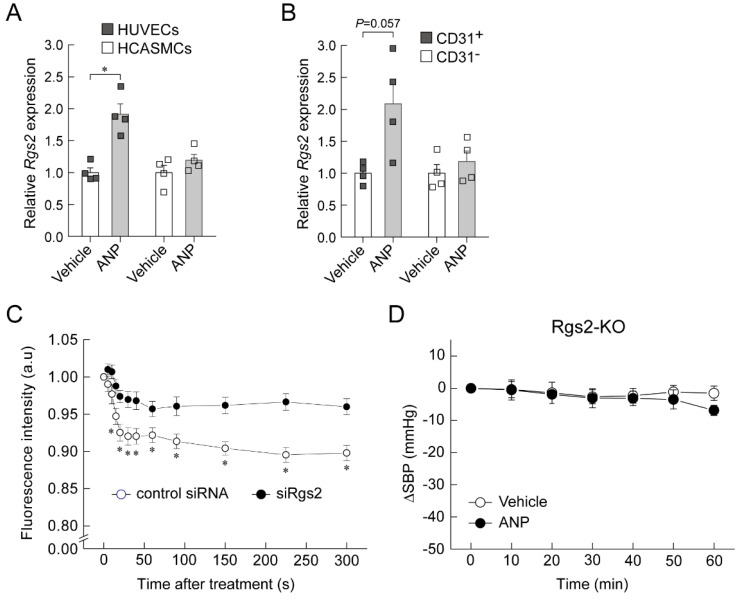
RGS2 plays a key role in the hypotensive effect of ANP infusion. (**A**) ANP increased *Rgs2* mRNA expression in human umbilical vein endothelial cells (HUVECs) but not in human coronary artery smooth muscle cells (HCASMCs). HUVECs and HCASMCs were treated with vehicle (PBS) or ANP for 60 min. mRNA levels were normalized to those of GAPDH. Values are expressed relative to vehicle-treated cells. * *p* < 0.05. (**B**) ANP increased *Rgs2* mRNA expression in CD31^+^ cells but not in CD31^−^ cells. After 48 h of continuous administration of vehicle (saline) or ANP (0.2 μg/kg/min), the *Rgs2* mRNA levels were evaluated in CD31^+^ cells and CD31^−^ cells. (**C**) RGS2 plays a key role in ANP-mediated hyperpolarization in HUVECs. Changes in the membrane potential in HUVECs transfected with control siRNA or *Rgs2* siRNA (siRgs2) were evaluated. DiBAC4(3)-loaded HUVECs were treated with ANP. Values are expressed relative to the intensity of the control siRNA-transfected cells at time 0. * *p* < 0.05 vs. siRgs2. (**D**) ANP infusion failed to decrease systolic blood pressure (SBP) in RGS2 knockout (Rgs2-KO) mice. Intravenous infusion of ANP (10 μg/kg/min) did not significantly lower SBP compared with the vehicle infusion. This figure is a reconstruction of the figure in our original article [37].

**Figure 7:**
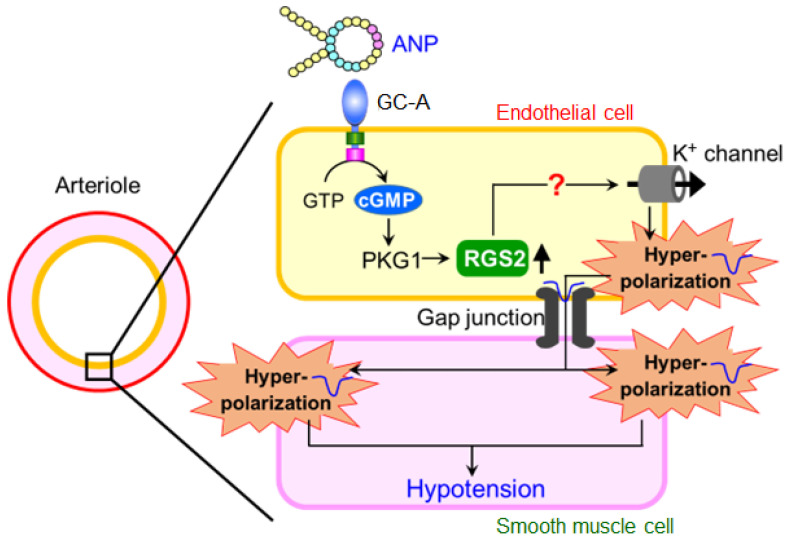
Proposed mechanism underlying the acute blood pressure decrease induced by the administration of ANP. The hypotensive effect of ANP is independent of nitric oxide and can be caused, at least in part, by endothelial GC-A-mediated, RGS2-dependent endothelial hyperpolarization. The exact mechanism of the production of the RGS2-induced endothelium-derived hyperpolarizing factor and subsequent hyperpolarization of smooth muscle cells requires further investigation. This figure is a reconstruction of the figure in our original article [37].

RGS2 is a GTPase-activating protein for the G_q/11_α and G_i/o_α subunits [42]. Rgs2-KO mice exhibit hypertension [43]. Recently, it has been reported that RGS2 deficiency in endothelial cells causes impairment of EDHF-dependent vascular relaxation [44]. In addition, we previously performed a DNA microarray analysis of ANP-stimulated HUVECs and found that the *Rgs2* gene expression was increased. Therefore, we hypothesized that RGS2 is involved in the hypotensive effect of ANP infusion. Quantitative real-time PCR analysis confirmed that ANP stimulation significantly increased *Rgs2* mRNA expression in HUVECs, but not in HCASMCs (Figure 6A) [37]. We also examined whether the infusion of ANP increased the endothelial *Rgs2* mRNA expression in wild-type mice. The *Rgs2* mRNA expression tended to increase in lung CD31^+^ cells, but not in CD31^−^ cells, in ANP-infused wild-type mice relative to vehicle-infused mice (Figure 6B) [37]. Therefore, it is likely that the increase in *Rgs2* mRNA expression by ANP stimulation is endothelium specific. We also investigated the role of RGS2 in ANP-mediated hyperpolarization in HUVECs. Transfection with siRNA targeting *Rgs2* (si*Rgs2*) significantly blocked the ANP-mediated decrease in DiBAC4 fluorescence (Figure 6C) [37]. Finally, we used Rgs2-KO mice to examine whether RGS2 is required for ANP-mediated hypotension. There were no significant differences in systolic blood pressure between vehicle- and ANP-infused Rgs2-KO mice (Figure 6D), indicating that RGS2 is necessary for the hypotensive effect of ANP infusion. Figure 7 demonstrates the proposed mechanism underlying the acute decrease in blood pressure induced by ANP administration.

## 7. Long-Term Regulation of Blood Pressure by ANP

**Figure 8:**
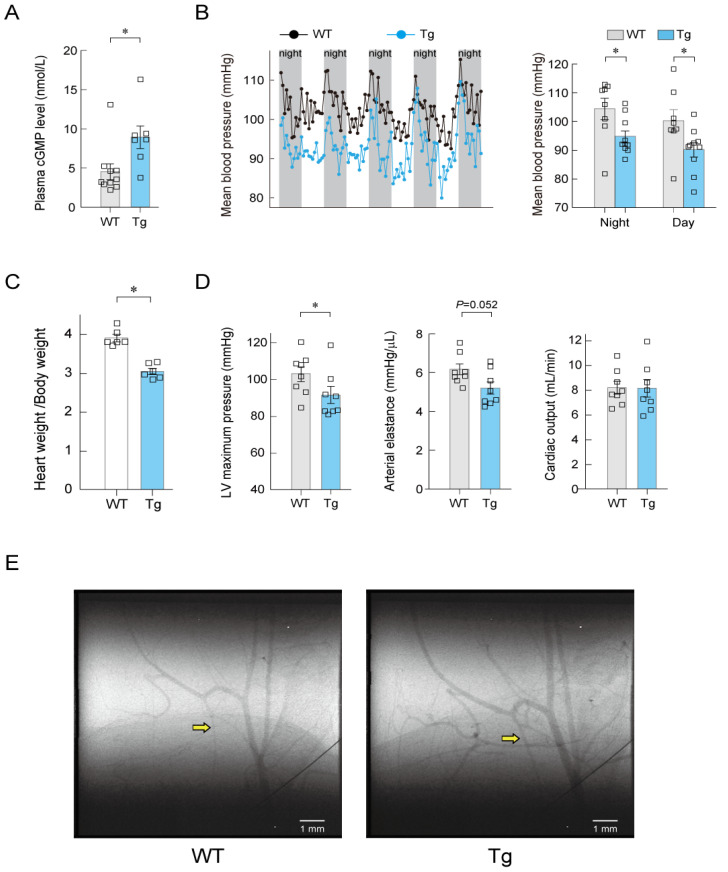
Endothelium-specific overexpression of GC-A results in hypotension and vasodilation in mice. (**A**) The plasma cyclic GMP level was higher in endothelial-cell-specific GC-A-overexpressing (Tg) mice than in wild-type (WT) mice. (**B**) The mean blood pressure, as determined by radiotelemetry, was significantly lower in the Tg mice than in the WT mice. (**C**) The heart weight to body weight ratio was significantly smaller in the Tg mice than in the WT mice. (**D**) Hemodynamic analysis revealed that the left ventricular (LV) maximum pressure and arterial elastance were lower in Tg mice than in the WT mice, but the cardiac output was comparable between the two types of mice. (**E**) Angiographic images from a WT mouse and a Tg mouse. Yellow arrows indicate a vessel that is dilated in the Tg relative to the WT. * *p* < 0.05. This figure is a reconstruction of the figure in our original article [37].

When studying long-term blood pressure regulation by ANP, the phenotypes of genetically engineered mice are informative. GC-A-deficient mice exhibit hypertension and increased heart weight [45,46,47]. ANP-deficient mice also develop elevated blood pressure and increased heart weight [48]. Conversely, Field’s group reported in 1994 that mice overexpressing ANP in the liver, whose ANP blood levels were approximately five times higher than in wild-type mice, showed a lower blood pressure, lower total peripheral vascular resistance, and lower heart weight than the wild-type mice [49]. The cardiac output was comparable in the ANP-overexpressing and wild-type mice [49], suggesting that the cause of the decreased blood pressure was reduced vascular resistance. If this is so, which cellular GC-A receptor primarily contributes to long-term blood pressure regulation by ANP? As mentioned in the introduction, SMC-GC-A-KO mice do not exhibit hypertension [17], whereas EC-GC-A-KO mice do [18]. This suggests that the endothelial ANP-GC-A system is of greater importance than the smooth muscle ANP-GC-A system for long-term blood pressure regulation. It has been reported that hypertension in EC-GC-A-KO mice may be caused by increased cardiac output [18]. Does this mean that changes in vascular resistance do not contribute to long-term blood pressure regulation by vascular endothelial GC-A? To answer this question, we generated endothelium-specific GC-A–overexpressing mice (EC-GC-A-Tg) using the *Tie2* promoter and enhancer. The plasma cyclic GMP concentration in EC-GC-A-Tg mice was approximately twice that in wild-type mice (Figure 8A) [37]. Figure 8B shows the mean blood pressure results measured by telemetry. In both dark and light phases, the mean blood pressure in EC-GC-A-Tg mice was significantly lower than in the wild-type mice [37]. The ratio of heart weight to body weight was significantly reduced in EC-GC-A-Tg mice compared with wild-type mice (Figure 8C) [37]. To examine why the blood pressure of EC-GC-A-Tg mice was significantly lower than that of wild-type mice, the hemodynamic parameters of both mouse types were evaluated by pressure–volume catheterization. Left ventricular maximum pressure was significantly lower and arterial elastance tended to be lower in EC-GC-A-Tg mice than in wild-type mice, but the cardiac output was comparable between the two (Figure 8D) [37]. Together, these findings suggest that the cause of hypotension in EC-GC-A-Tg mice is a decrease in vascular resistance.

To confirm that dilation of the peripheral arteries was responsible for the lower vascular resistance observed in EC-GC-A-Tg mice, we performed microangiography at SPring-8 (Hyogo, Japan) [37]. Femoral arteries and the femur were visualized. Angiographic images of EC-GC-A-Tg mice and wild-type mice are shown in Figure 8E. Vessels are clearly more dilated in EC-GC-A-Tg mice than in wild-type mice, especially on the distal side. Together, these findings suggest that the overexpression of GC-A in the endothelium leads to dilation of the small arteries and arterioles, resulting in hypotension without an altered cardiac output.

## 8. Summary and Conclusions

The mechanisms underlying ANP-induced hypotension and long-term blood pressure regulation by ANP are not fully understood. We hypothesized that endothelial ANP-GC-A signaling plays an important role in blood pressure regulation. We found that intravenous ANP infusion resulted in a significant decrease in systolic blood pressure in SMC-GC-A-KO mice, but not in EC-GC-A-KO mice. Interestingly, ANP infusion significantly reduced systolic blood pressure in Nos3-KO mice. These findings indicate that vascular endothelial GC-A plays a central role in hypotension induced by acute intravenous ANP infusion, and that the endothelial nitric oxide pathway is not involved. We also found that blood pressure in EC-GC-A-Tg mice was significantly lower than that in wild-type mice, and that the diameter of resistance arteries was significantly larger in the former than in the latter. These results indicate that vascular endothelial GC-A is also important in the long-term regulation of blood pressure and vascular tone. These are new findings that contradict the previously accepted mechanism of the action of ANP.

Endothelial cells control vascular tone not only by releasing nitric oxide, but also by hyperpolarizing smooth muscle cells via EDHF [39,40,41]. EDHF plays an important role in the regulation of vascular tone in small arteries and arterioles [39,40,41]. In the study we summarized here, ANP hyperpolarized HUVECs, but this process was inhibited by a non-selective potassium channel blocker, suggesting that endothelial hyperpolarization via potassium channels may be involved in the vasodilatory effect of ANP. Recently, Osei-Owusu et al. demonstrated that endothelial-specific deletion of *Rgs2* markedly inhibited EDHF-dependent relaxation of mouse mesenteric arteries [42,44]. We found that ANP significantly increased *Rgs2* mRNA expression levels in HUVECs and that ANP-mediated hyperpolarization of HUVECs was abolished under *Rgs2* siRNA transfection. Furthermore, we found that ANP infusion failed to decrease blood pressure in Rgs2-KO mice. Together, these findings suggest that endothelial RGS2 plays an important role in ANP-mediated hypotension. Further studies are needed to elucidate the exact mechanisms of the ANP-induced increase in *Rgs2* mRNA expression in endothelial cells and of RGS2-induced endothelial hyperpolarization. More research is also required on the significance of RGS2 in the long-term control of blood pressure by ANP.

In conclusion, we propose that ANP-induced hypotension is caused, at least in part, by GC-A-mediated, RGS2-induced hyperpolarization of endothelial cells, and further studies are needed to clarify the molecular mechanism through which long-term GC-A overexpression in endothelial cells leads to hypotension.

## 9. Future Directions

This review discusses the molecular mechanisms of acute and long-term blood pressure regulation by ANP. This is a simple topic, but one that is surprisingly difficult to investigate. ANP is a hormone produced in the heart, and we believe its essence is the maintenance of organ blood flow. In fact, immunohistochemistry showed abundant GC-A expression in resistance vessels, and EC-GC-A-Tg mice exhibited dilation of the resistance vessels. These results are in excellent agreement with the statement that “ANP dilates resistance vessels” as expressed by Saito et al. in Circulation in 1987 [13]. Although ANP was discovered in 1984, its physiological and pharmacological effects remain to be clarified. We also expect that the worldwide clinical application of ARNI will provide new clinical evidence about ANP. We sincerely hope that young academics will enter ANP research.

## Data Availability

Data sharing not applicable.
